# Supplementary material for: Dachshund Homolog 1: Unveiling Its Potential Role in Megakaryopoiesis and Bacillus anthracis Lethal Toxin-Induced Thrombocytopenia
Source: Int J Mol Sci. 2024 Mar 7;25(6):3102. doi: 10.3390/ijms25063102 (PMC10970148; doi:10.3390/ijms25063102)
Supplement: Supplementary file 1 [file ijms-25-03102-s001.zip › ijms-2867285-supplementary.pdf]

Supplementary Materials

Dachshund homolog 1: Unveiling its potential role in megakaryopoiesis and *Bacillus anthracis* lethal toxin-induced thrombocytopenia

Guan-Ling Lin[1,2,3], Hsin-Hou Chang[1,3], Wei-Ting Lin[3], Yu-Shan Liou[3], Yi-Ling Lai[3], Min-Hua Hsieh[3], Po-Kong Chen[1], Chi-Yuan Liao[4], Chi-Chih Tsai[4], Tso-Fu Wang[5,6,7], Sung-Chao Chu[5,6,7], Jyh-Hwa Kau[8], Hsin-Hsien Huang[8], Hui-Ling Hsu[8], Der-Shan Sun[1,3*]

[1]Institute of Medical Sciences, Tzu-Chi University, Hualien, Taiwan, Republic of China.

[2]Integration Center of Traditional Chinese and Modern Medicine, Hualien Tzu Chi Hospital, Buddhist Tzu Chi Medical Foundation, Hualien, Taiwan, Republic of China.

[3]Department of Molecular Biology and Human Genetics, Tzu-Chi University, Hualien, Taiwan, Republic of China.

[4]Department of Obstetrics and Gynecology, Mennonite Christian Hospital, Hualien, Taiwan, Republic of China.

[5]Department of Hematology and Oncology, Hualien Tzu Chi Hospital, Buddhist Tzu Chi Medical Foundation, Hualien, Taiwan, Republic of China.

[6]Department of Medicine, College of Medicine, Tzu Chi University, Hualien, Taiwan, Republic of China.

[7]Buddhist Tzu Chi Stem Cells Center, Hualien Tzu Chi Hospital, Buddhist Tzu Chi Medical Foundation, Hualien, Taiwan, Republic of China.

[8]Institute of Preventive Medicine, National Defense Medical Center, Taipei, Taiwan, Republic of China.

*Corresponding author:

Der-Shan Sun, Ph.D.

Department of Molecular Biology and Human Genetics, Tzu-Chi University,

No. 701, Section 3, Zhong-Yang Road, Hualien 97004, Taiwan, Republic of China.

Phone: 886-3-8565301 ext. 2681 FAX: 886-3-8561422

Email: dssun@mail.tcu.edu.tw

Methods

Cell viability assay

J774A.1 cells were seeded onto 96-well microtiter plates at a density of 5×10^4 cells per well and cultured in 100 µl DMEM medium supplemented with 1% heat-inactivated fetal bovine serum for 16 hours. The cells were then treated with a lethal toxin (LT) isolated from *Bacillus anthracis* with serial dilution from stock (1.55 mg/ml for the first and 0.36 mg/ml for the second batch). Untreated cells were used as negative controls. Cell viability was assessed after a 3-hour incubation period using the WST-1 assay (Roche, Mannheim, Germany) for the first batch of LT or the Cell Counting Kit-8 (CCK-8) kit (Sigma-Aldrich, St. Louis, MO, USA) for the second batch of LT. For the WST-1 assay, WST-1 reagent was added to each culture well at a final dilution of 1:20 in RPMI-1640 medium without phenol red (Gibco). The plate was then incubated for 30 minutes at 37°C. For the CCK-8 assay, 10 µl CCK-8 reagent directly was added directly into the well and incubated for 2 hours at 37°C. After incubation, the absorbance was measured at a wavelength of 450 nm using an ELISA reader (Rosys Anthos HT1, Germany). The reference wavelength used was 620 nm for the WST-1 assay and 630 nm for the CCK-8 assay. The viability ratio was calculated as the absorbance of the treated cells (A450) divided by the absorbance of the untreated cells (A450).

Figure legends

Figure S1. Cytotoxicity test of the first batch of *Bacillus anthracis* lethal toxin on J774A.1 cells. J77A.1 cells were untreated or treated with *B. anthracis* LT (1.55 mg/ml) at various dilutions–500-fold, 1,000-fold, 2,000-fold, 4,000-fold, and 8,000-fold for 3 hours. Cell viability was assessed using the WST-1 kit, with triplicates performed for each condition. The cell viability in untreated groups was considered as 100%. Data are presented as mean ± the standard error of the mean (SEM). The statistical significance is indicated by $*P$ value < 0.05, $**P$ value < 0.01, compared with the untreated groups.

Figure S2. Cytotoxicity test of the second batch of *Bacillus anthracis* lethal toxin on J774A.1 cells. J774A.1 cells were either untreated or exposed to LT at a concentration of 0.36 mg/ml, with subsequent dilutions of 250-fold, 500-fold, 750-fold, 1,000-fold, 2,000-fold, 4,000-fold, and 8,000-fold for 3 hours. Cell viability was assessed using the CCK-8 kit, with three replicates conducted for each condition. The cell viability in untreated groups was set as 100%. The data are presented as mean ± SEM. Statistical significance is denoted by $*P < 0.05$, $**P < 0.01$, compared with the untreated groups.

Figure S3. The dynamic changes in live and dead cell. A total of 1×10^6 HEL cells per 24-well culture dish were either untreated or treated with 0.01% DMSO (vehicle), TPA, LT, and a combination of TPA and LT. The Trypan blue exclusion assay was conducted to enumerate live (A) and dead cells (B) at each time point (24 hr, 48 hr, and 72 hr).

Figure S4. Effect of LT and U0126 on megakaryocytic differentiation. HEL cells were either left untreated or treated with LT, TPA (10^{-8} M), TPA combined with LT, and TPA combined with U0126 (10 μM) for three days. HEL cells treated with 0.01% DMSO served as the vehicle control. The percentage of cells expressing the megakaryocytic-specific surface marker CD61 was monitored (A) and analyzed (B) using flow cytometry.

Figure S5. Unedited images of Western blot in Fig. 2B. HEL cells were subjected to different treatments: 0.01% DMSO (lane 1), TPA (lane 2), and TPA combined with LT (lane 3) for 72 hours. The left panel presents the original images incubated with antibodies against DACH1 (ab31588 and ab176718, Abcam) (A) and subsequently re-probed with antibodies against β-actin (B). The right panel displays the Western blot Ponceau S stained membrane before primary antibody treatment (C) and the Western blot membrane after antibody treatment (D). The red box highlights the cropped blots representing identical proteins used in Fig. 2B. The red arrow indicates the DACH1 signal developed with the antibody against DACH1 (ab176718). The pre-stained protein markers in the image are obtained from Thermo Page Ruler.

Figure S6. Western blot with DACH1 antibody (ab31588, Abcam). HEL cells were treated with 0.01% DMSO (lane 1), TPA (lane 2), and TPA combined with LT (lane 3) for 72 hours. Membranes were incubated with antibodies against DACH1 (A) and β-actin (B) on different membranes. The right panel displays the Western blot membranes after antibody treatment, and the positions of the protein markers (Bio-Rad) are indicated by Ponceau S staining after transfer.

Figure S7. Western blot with DACH1 antibody (ab31588, Abcam). HEL cells were treated with 0.01% DMSO (lane 1), TPA (lane 2), and TPA combined with LT (lane 3) for 72 hours. Membranes were incubated with antibodies against DACH1 (A) and β-actin (B) on different membranes. The right panel displays the Western blot membranes after antibody treatment, and the positions of the protein markers (top: GeneDireX, bottom: Bio-Rad) are indicated by Ponceau S staining after transfer.

Figure S8. Effect on megakaryocytic differentiation after DACH1 knockdown by shRNA. HEL cells were transfected with viruses carrying shLacZ, #4 shDACH1, or #5 shDACH1, followed by treatment with TPA for three days to induce megakaryocytic differentiation. Subsequently, the cells were stained with PI, and their DNA contents were analyzed using flow cytometry.

Figure S9. Unedited images of Western blot in Fig. 4A. HEL cells were treated with 0.01% DMSO (lane 1), TPA (lane 2), and TPA combined with LT (lane 3) for 72 hours. Membranes were incubated with antibodies against MEK-1 (A) and MEK-2 (sc-524, Santa Cruz) (B), and subsequently re-probed with antibodies against β-actin. The red box indicates the regions where the blots were cropped, featuring identical proteins, as presented in Fig. 4A. The right panel displays the Western blot membranes after antibody treatment (top) and the Western blot Ponceau S stained membrane before primary antibody treatment (down). Pre-stained protein markers labeled in the image include Thermo Page Ruler (top) and GeneDireX (bottom).

Figure S10. Western blot with another MEK-2 antibody. HEL cells were treated with 0.01% DMSO (lane 1), TPA (lane 2), and TPA combined with LT (lane 3) for 72 hours. Membranes were incubated with antibodies against MEK-2 (sc-13159, Santa Cruz), and subsequently re-probed with antibodies against β-actin. The red arrow indicates the MEK-2 signal developed with the antibody against MEK-2 (sc-13159). The right panel displays the Western blot Ponceau S stained membrane before primary antibody treatment. The pre-stained protein markers in the image are obtained from Thermo Page Ruler.

Figure S11. Unedited images of Western blot in Fig. 4B. HEL cells were treated with 0.01% DMSO (lane 1), TPA (lane 2), and TPA combined with LT (lane 3) for 72 hours. Membranes were incubated with antibodies against phosphorylated ERK (p-ERK) and subsequently re-probed with antibodies against EPK. The red box indicates the regions where the blots were cropped, featuring identical proteins, as presented in Fig. 4B. The right panel shows the Western blot membranes with protein markers labeled. The positions of the protein markers (right side, Bio-Rad, left side: Pre-stained protein markers-Thermo Page Ruler) are indicated by Ponceau S staining after transfer.

Figure S12. Dynamic change in the percentage of $CD61^+$ cells during the CD34–MK *in vitro* differentiation model. The umbilical cord blood-derived $CD34^+$ cells were cultured on umbilical cord-derived mesenchymal stem cells for expansion. Subsequently, these cells were cultured in a differentiation medium to initiate megakaryocytic differentiation for 16 days. Cell size (forward scatter: FSC) and cell granularity (side scatter, SSC) were assessed

every two days using flow cytometry (A). Two distinct cell populations, designated as R1 and R2 regions, were identified based on their size and granularity. The percentages of $CD61^+$ cells within the R1 region were monitored using flow cytometry every two days (B).

Fig-S1

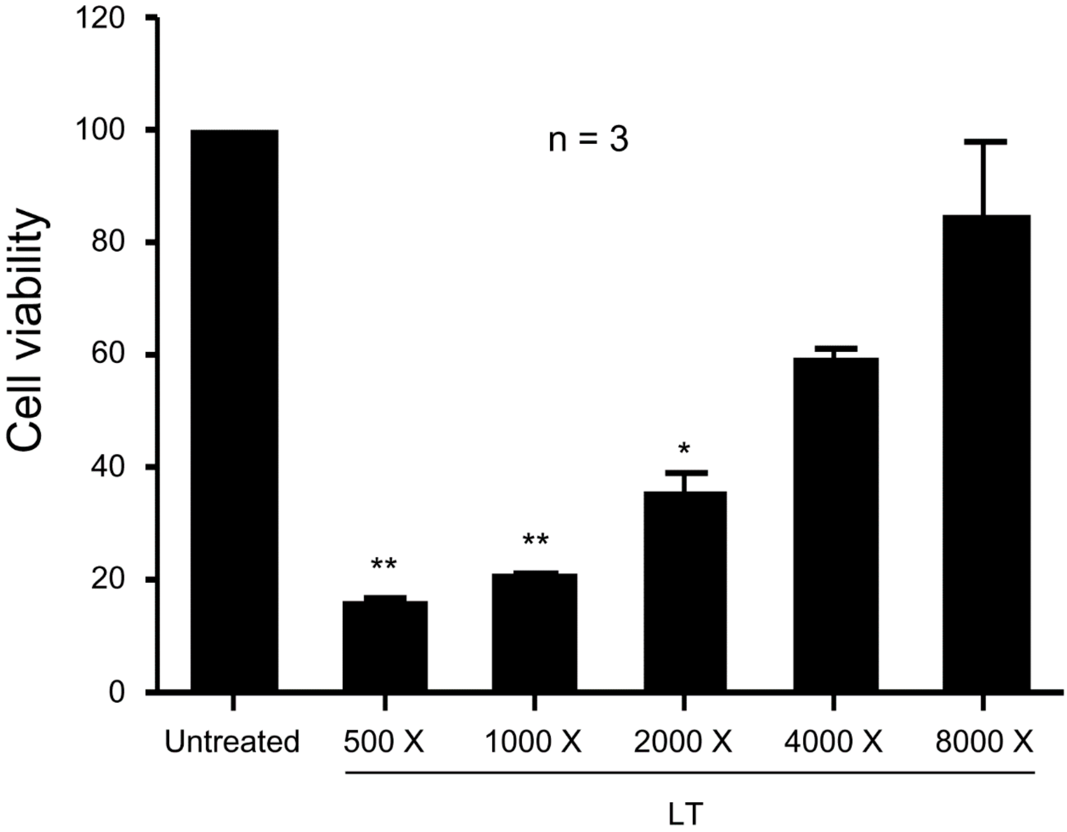
120
100
80
60
40
20
0
Cell viability
n = 3
**
**
*
Untreated
500 X
1000 X
2000 X
4000 X
8000 X
LT

Fig-S2

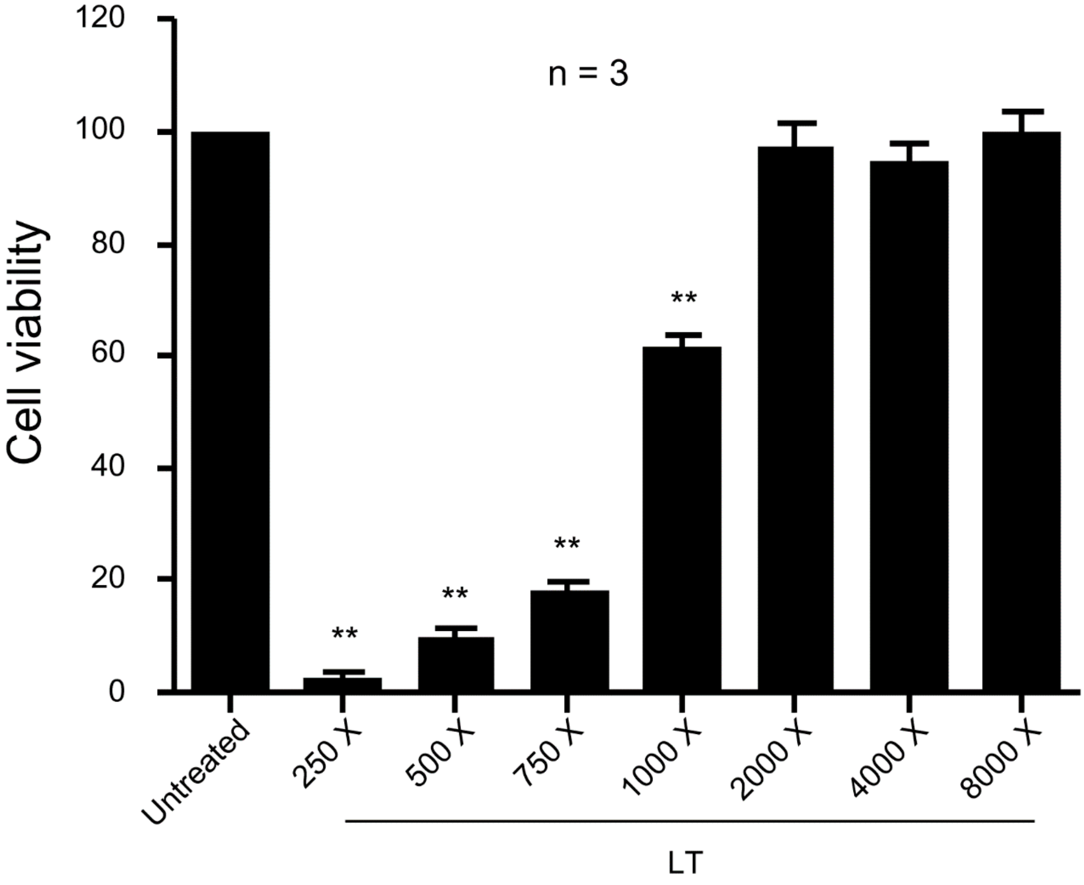

n = 3
Cell viability
120
100
80
60
40
20
0
**
**
**
**
Untreated
250 X
500 X
750 X
1000 X
2000 X
4000 X
8000 X
LT

Fig-S3

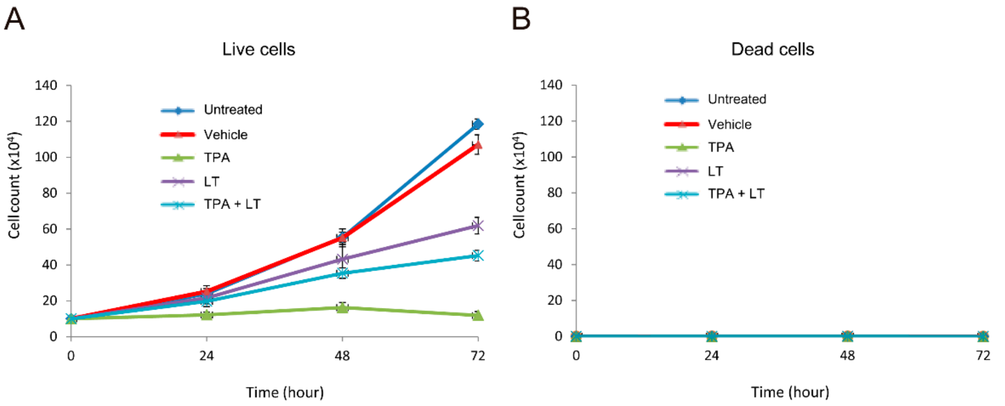
A
Live cells
Cell count (x10⁴)
140
120
100
80
60
40
20
0
0
24
48
72
Time (hour)
Untreated
Vehicle
TPA
LT
TPA + LT
B
Dead cells
Cell count (x10⁴)
140
120
100
80
60
40
20
0
0
24
48
72
Time (hour)
Untreated
Vehicle
TPA
LT
TPA + LT

Fig-S4

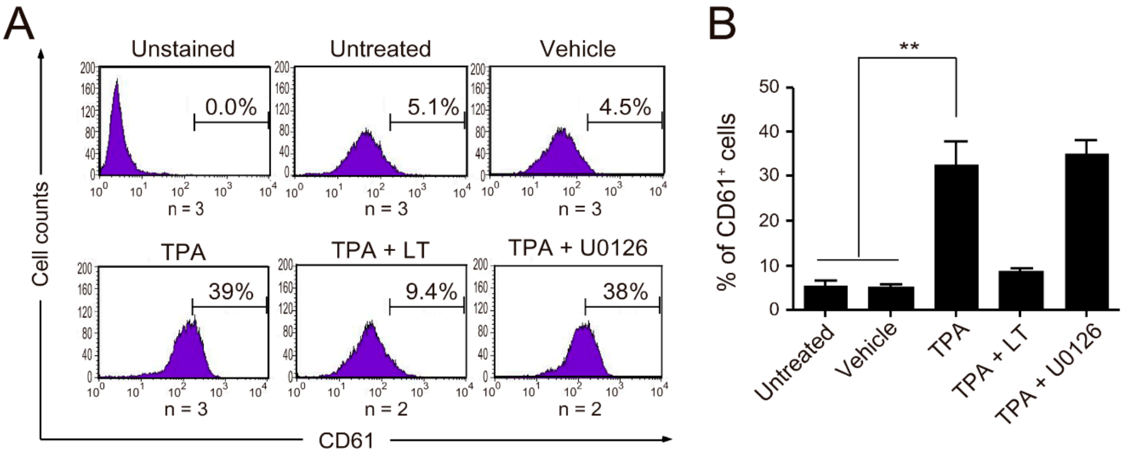
A
Unstained
Untreated
Vehicle
0.0%
5.1%
4.5%
n = 3
n = 3
n = 3
TPA
TPA + LT
TPA + U0126
39%
9.4%
38%
n = 3
n = 2
n = 2
Cell counts
CD61
B
**
% of CD61+ cells
Untreated
Vehicle
TPA
TPA + LT
TPA + U0126

Fig-S5

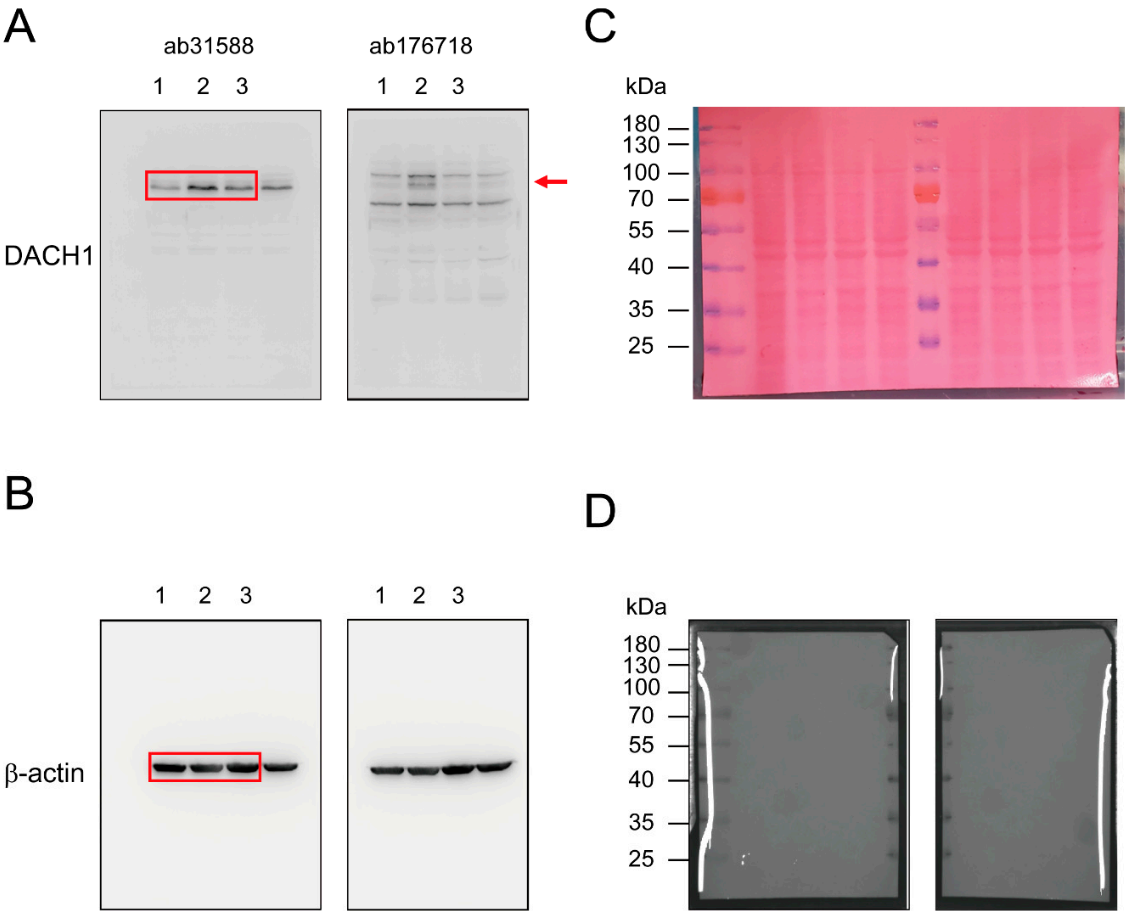
A
ab31588
ab176718
1 2 3
1 2 3
DACH1
B
1 2 3
1 2 3
β-actin
C
kDa
180
130
100
70
55
40
35
25
D
kDa
180
130
100
70
55
40
35
25

Fig-S6

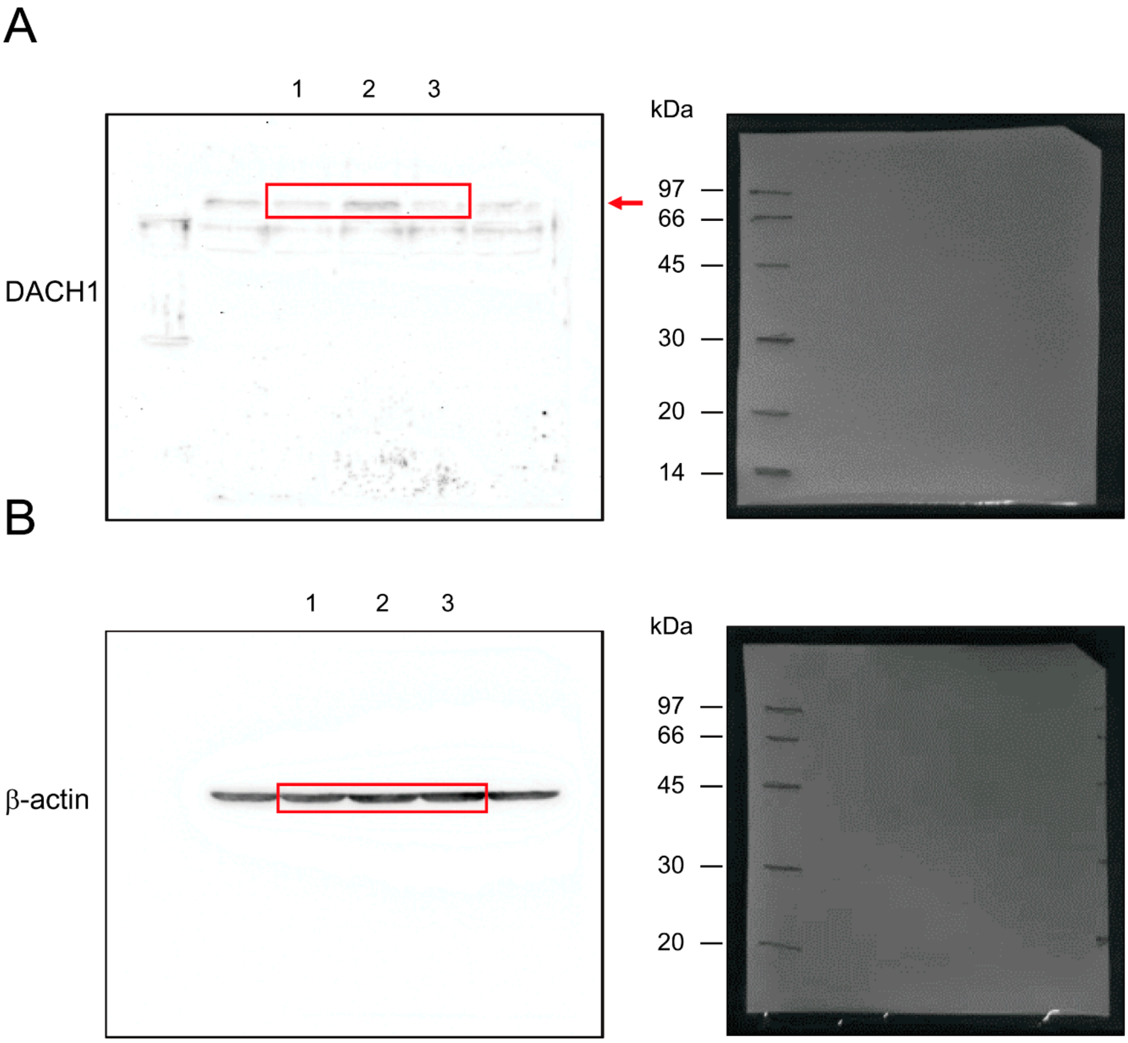

Fig-S7

A

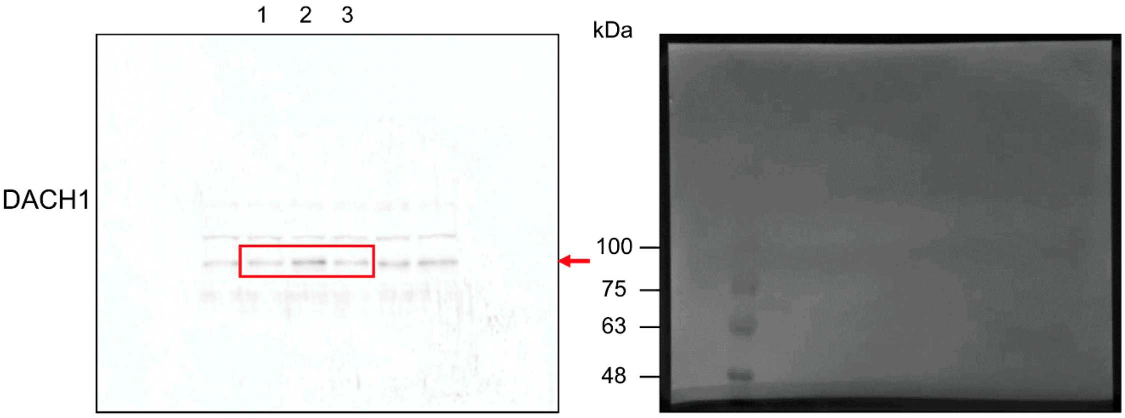

B

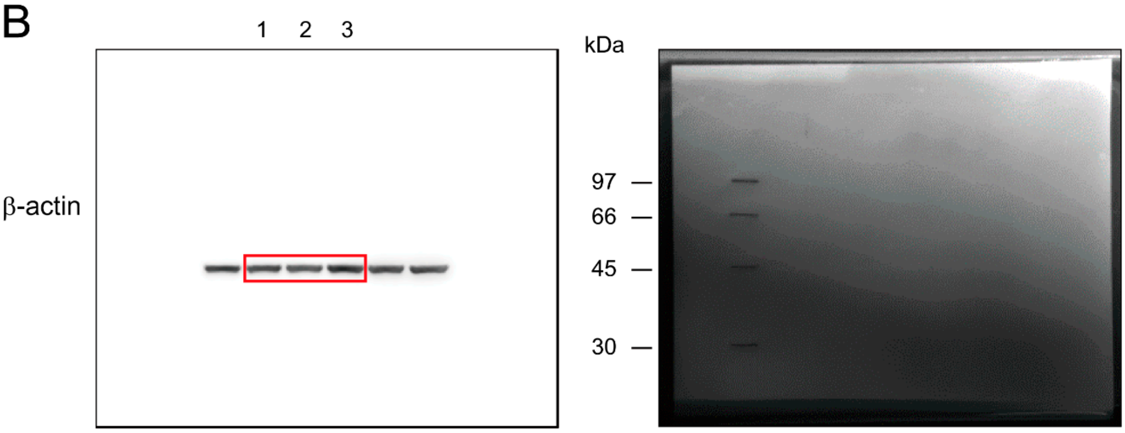

Fig-S8

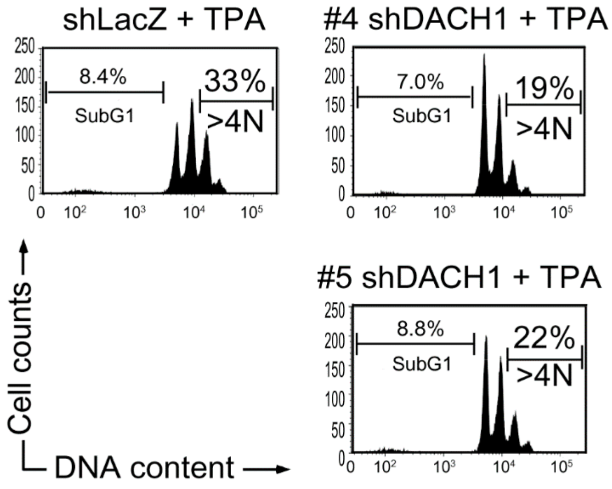
shLacZ + TPA
8.4%
SubG1
33%
>4N
#4 shDACH1 + TPA
7.0%
SubG1
19%
>4N
#5 shDACH1 + TPA
8.8%
SubG1
22%
>4N
Cell counts
DNA content

Fig-S9

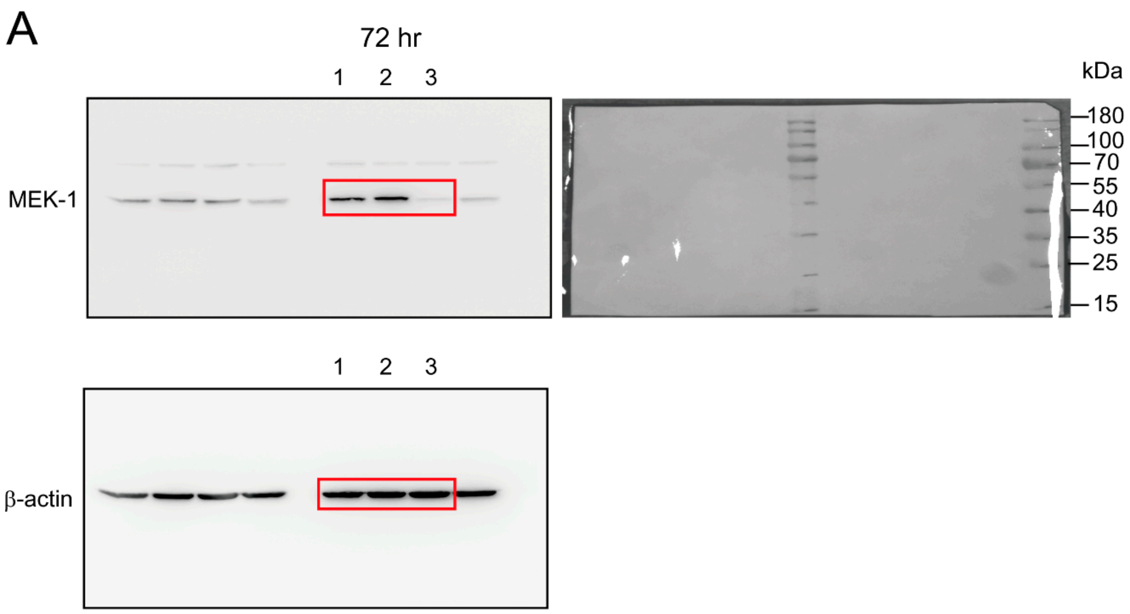
A
72 hr
1 2 3
MEK-1
kDa
180
100
70
55
40
35
25
15
1 2 3
β-actin

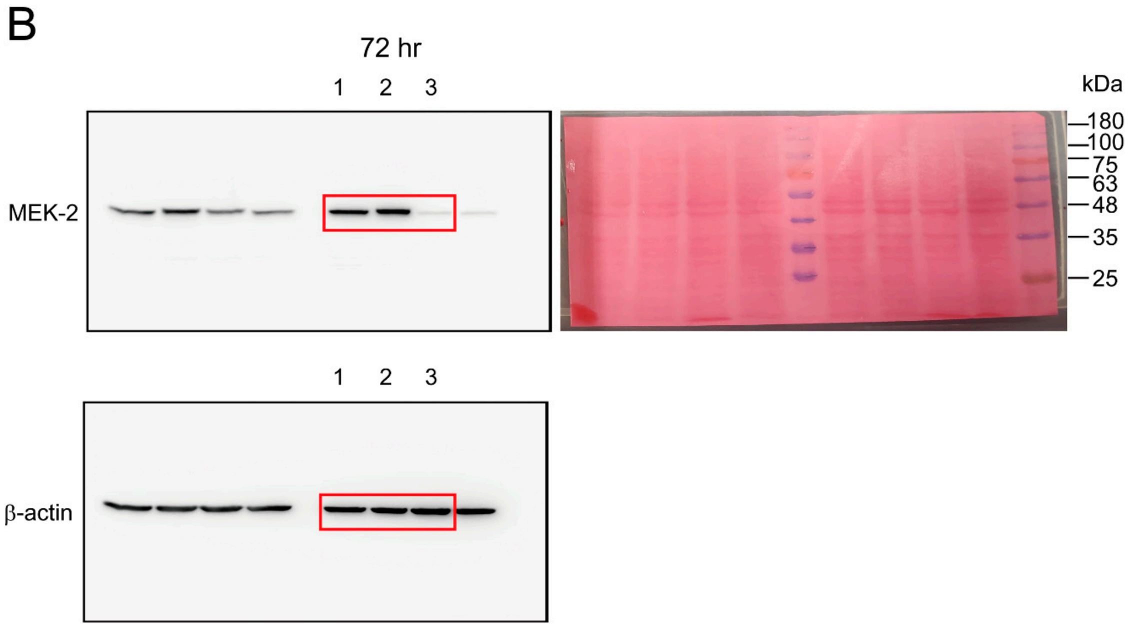
B
72 hr
1 2 3
MEK-2
kDa
180
100
75
63
48
35
25
1 2 3
β-actin

Fig-S10

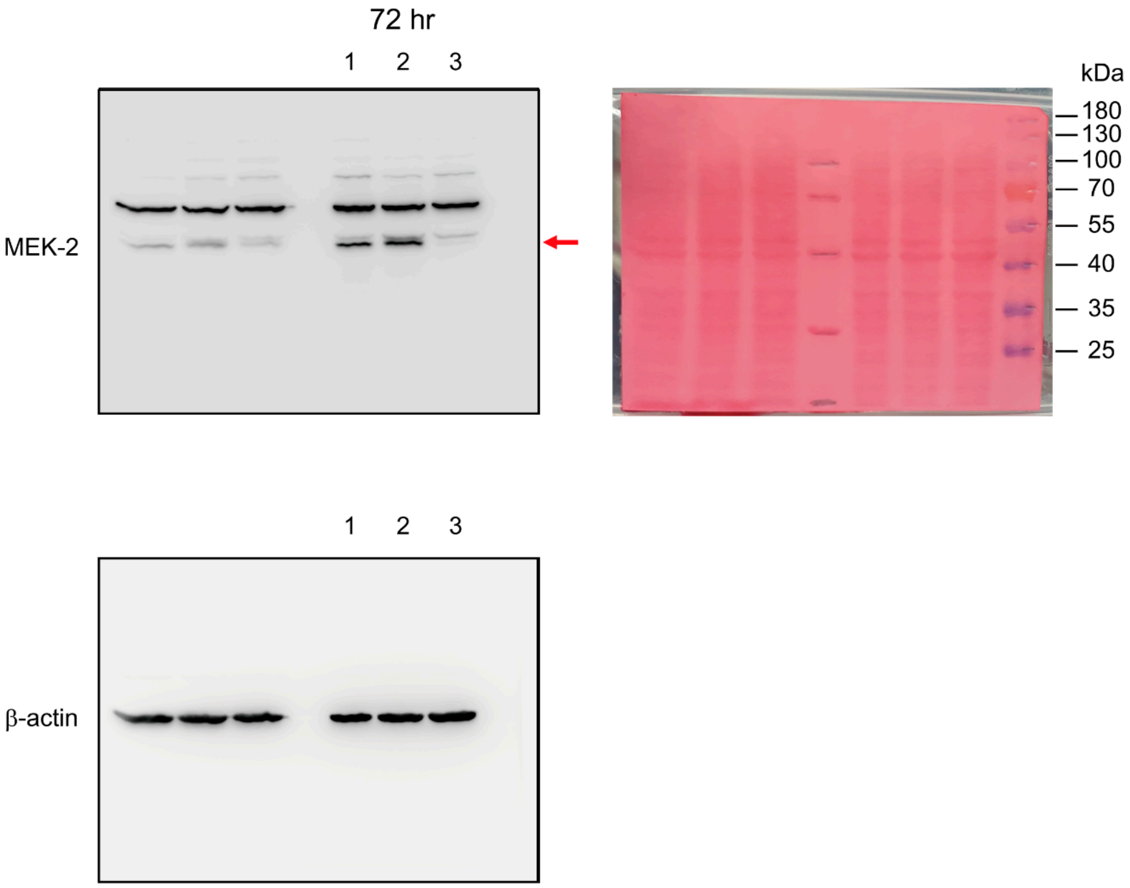

Fig-S11

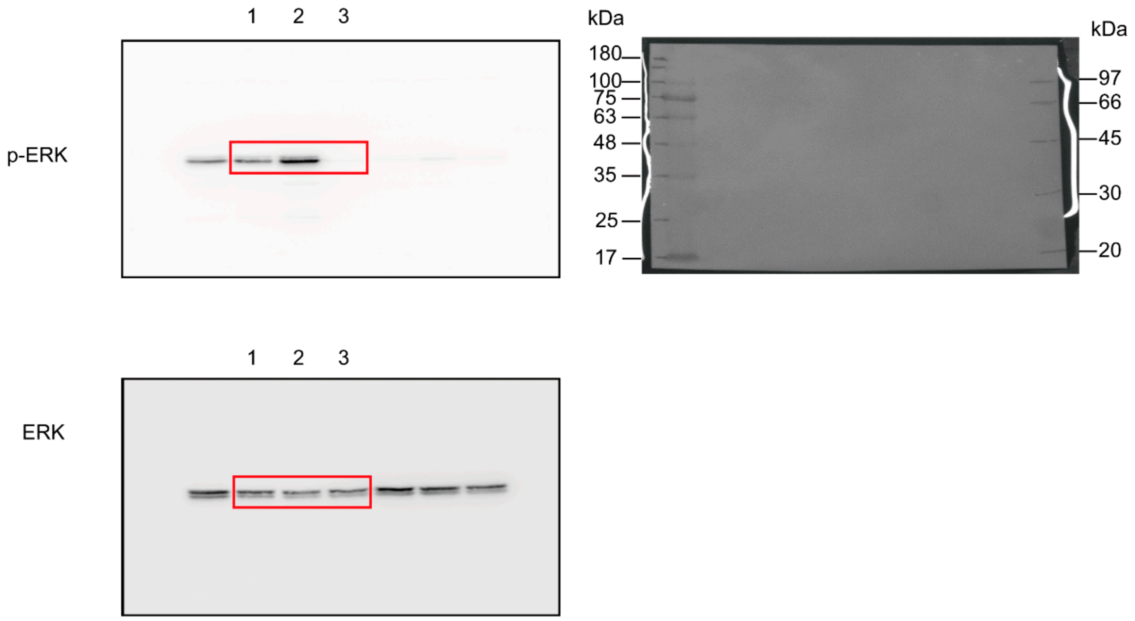

Fig-S12

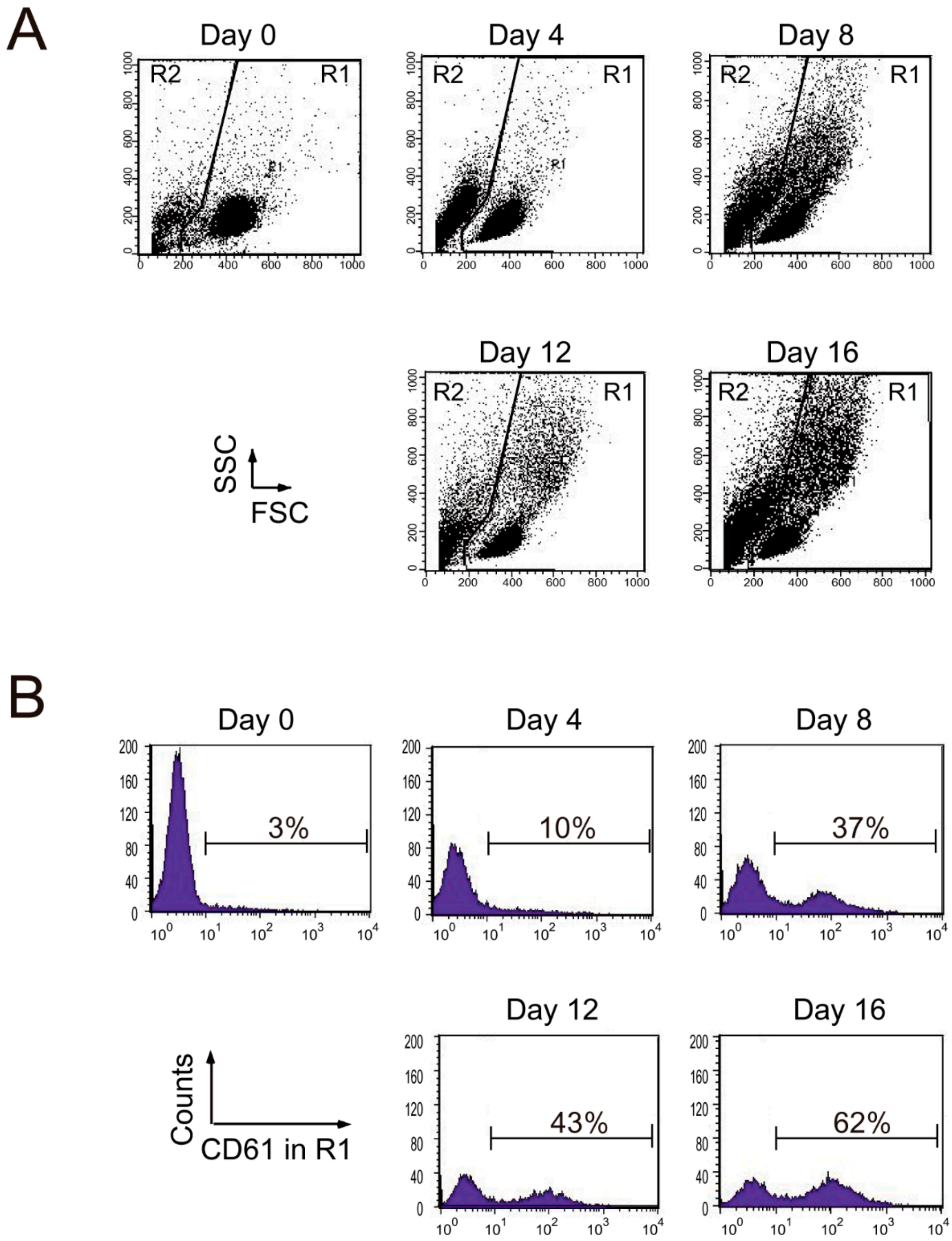
A
Day 0
Day 4
Day 8
Day 12
Day 16
R2
R1
SSC
FSC
B
3%
10%
37%
43%
62%
Counts
CD61 in R1

Table S3

Sequences of primers for qRT-PCR

Gene	Primer Sequence
DACH1	Forward: 5’-TCAGCCTACCTCCAGCATCT-3’
	Reverse: 5’-TGAGGGGCTATCAGGAACAC-3’
FOSB	Forward: 5’-ATCCCACATTTCCATGGTGT-3’
	Reverse:5’-GAGGCCAGAAATTCCAATCA-3’
ZFP36L1	Forward: 5’-GACCTTCGCGACACACCAGA-3’
	Reverse: 5’-GCTGGTTCTGGTGGAACTTG-3’
RUNX1	Forward: 5’-TGAAGAACCAGGTTGCAAGA-3'
	Reverse: 5’-TTTTGATGGCTCTGTGGTAGG-3’
FLI1	Forward:5’-TGCTGTTGTCACACCTCAGTTA-3’
	Reverse: 5’-ATTGCCCCAAGCTCCTCTT-3’
AHR	Forward:5’-CACGAGAGGCTCAGGTTATCA-3’
	Reverse: 5’-CCAAGTCCATCGGTTGTTTT-3’
GATA1	Forward: 5’-TGAGGCCTACAGACACTCCC-3’
	Reverse: 5’-CACACAGTTGAGGCAGGGTA-3’
NFE2	Forward: 5’-CCACTTCCTCCACCACCTTA-3’
	Reverse: 5’-TCGGATTCTGGGTCTTCTTG-3’
GFI1B	Forward: 5’-AGGCTCACACCTACCACCAG-3’
	Reverse: 5’-GCTCTCGTTTGAGGTTGGTC-3’
GAPDH	Forward: 5’-CGAGATCCCTCCAAAATCAA-3’
	Reverse: 5’-TTCACACCCATGACGAACAT-3

Table S4

Oligonucleotide sequences of shRNA clones

Name	Oligo Sequence
shLacZ (TRCN0000072233)	
5’-CCGGCGACCACGCAAATCAGCGATTCTCGAGAATCGCTGATTTGCGTGGTCGTTTTTG-3’	
shDACH1#4 (TRCN0000118090)	
5’-CCGGACTCTCACATCATGCCGCATTCTCGAGAATGCGGCATGATGTGAGAGTTTTTTG-3’	
shDACH1#5 (TRCN0000118091)	
5’-CCGGGCTGTTGAAAGTTGCCATAGACTCGAGTCTATGGCAACTTTCAACAGCTTTTTG-3’	

Underlined sequences represent the target sequences of the corresponding gene.
